# Technological Innovation, Sustainable Green Practices and SMEs Sustainable Performance in Times of Crisis (COVID-19 pandemic)

**DOI:** 10.1007/s10796-022-10250-z

**Published:** 2022-02-09

**Authors:** Mansour Naser Alraja, Rabia Imran, Basel M. Khashab, Mahmood Shah

**Affiliations:** 1 Department of Management Information Systems, College of Commerce and Business Administration, Dhofar University, Salalah, Oman; 2 Department of Management, College of Commerce and Business Administration, Dhofar University, Salalah, Oman; 3 Department of Business, Northumbria University, Newcastle, UK; 4 Northumbria University - City Campus, Newcastle, UK

**Keywords:** Technological innovations, Green practices, Sustainable performance, Technology-organisation-environment, Resource based view

## Abstract

COVID-19 restrictions significantly affected SMEs, which have faced many challenges to their sustainability within this fragile new environment. This study proposes a holistic framework of sustainable performance by interrelating factors showing robust associations to produce this effect' for achieving sustainable performance in SMEs, through integrating the Technology Organisation Environment (TOE) and Resource Based View (RBV) models, to test how sustainable green practices can process the TOE factors when affecting sustainable performance. The research focuses on SMEs with worldwide employees and involves data collected from a range of different employees belonging to four different managerial levels. The process incorporated the analysis of 669 questionnaires to test the proposed hypotheses using structural equation modeling. Findings suggest that, together, TOE factors represent crucial inputs for green practices such as green training, green performance appraisal, that, in turn, mean critical processes lead to sustainable performance (output). Additionally, the findings should also inspire SMEs to focus even more effort on internal technological and organisational factors and to encourage an eco-friendly culture that would demand stakeholders adopt a more positive environmental stance.

## Introduction

The Covid-19 pandemic dynamically changed the business environment and placed sustainability as a concern for the business world. The business operations that challenge environmental stability and require a physical presence in any form started to face greater risks. Thus, as part of their social responsibility, organisations have adopted environmentally friendly and technologically advanced approaches that result in sustainable performance (Windolph et al., 2014; Zhu et al., 2019). In this regard, changing their product and process portfolios, being proactive, engaging in environmentally friendly practices and using technological innovations to sustain themselves has become a priority for organisations (Seuring & Gold, 2013). Technological innovations help cope with the pandemic, optimise the use of resources and meet sustainability goals (Klewitz & Hansen, 2014). The situation requires that organisations adopt new environmentally friendly technologies for sustainable processes and outcomes (Gallego-Álvarez et al., 2011).

Technological innovations can lead to environmentally friendly organisational processes. In the pandemic, innovative technologies with an environmental focus are required for sustainable performance. In this scenario, technological innovations help to optimally and effectively utilise scarce resources to create a competitive advantage and control operations remotely (El-Haddadeh, 2020; Klewitz & Hansen, 2014). The relationship between technological innovations and sustainable performance is complex and depends on numerous factors. One of the most integral factors is the type of innovation that affects business activities and influences specific resources (Sampson, 2007). This situation will increase the competitive advantage and lead to sustainable performance (Chege & Wang, 2020a). In the current pandemic scenario, all organisations invest in technological innovations to adopt sustainable green practices, such as green HRM, green supply chain, green innovation and green marketing, to achieve sustainable performance.

For more than a decade, developing countries have focused on Small and Medium Enterprises (SMEs) as a strong economic driver (Gbandi & Amissah, 2014). However, sustainability remains integral due to the slow success rate in the SME sector. Approximately half of SME startups fail within five years, especially in developing countries (Dalberg, 2011; Nikolić et al., 2019). A successful and sustainable SME requires the combination of essential contextual factors such as strong leadership and organisational practices. SMEs may start with enthusiasm and a strong vision, but in order to achieve the outlined performance goals and maintain a competitive advantage, the correct technological support and aligned processes must be in place (Alalawi, 2020; Ayyagari et al., 2007; Teresa Matriano and Firdouse Rahman Khan 2019). With COVID-19 restrictions and lockdowns put in place, the SME sector has faced a huge setback. Due to the requirement for a physical presence, this sector struggled with the new environment and finding ways to achieve sustainability.

The current study adopts the Technology-Organisation-Environment (TOE) model developed by Tornatzky and Fleischer (1990). In terms of coping with a dynamically changing environment, the model helps to explore the interplay between inputs, processes and outputs. The TOE framework is flexible and offers the right balance of internal and external drivers to help organisations effectively implement innovations (Aboelmaged, 2014). Few studies have used this framework in the context of sustainability. These studies include green IT initiatives (Hernandez & Ona, 2015), green supply chain (Hwang et al., 2016) and sustainable manufacturing practices (Aboelmaged, 2018). This model is helpful for developing a holistic framework of sustainable performance by combining several factors that can play their role in achieving sustainable performance. This study conceptualises technological innovation as input, sustainable green practices (such as green HRM, green supply chain, green innovation and green marketing) as process and sustainable performance as output. Additionally, the resource-based view (RBV) theory acts as the theoretical foundation for conducting the study.

The RBV proves relevant in this context for multiple reasons. Firstly, the theory has been used widely in studies of SMEs when measuring sustainability and competitive advantage as they are achievable by understanding the optimal utilisation of scarce and inimitable organisational resources (Barney, 1991; Gile et al., 2018). Secondly, the theory focuses on internal resources and emphasises that those resources can help formulate strategies for achieving targets, such as sustainability (Madhani, 2009). Should these resources prove inimitable and non-substitutable, the organisation would develop a competitive advantage, e.g. digitalisation, that would lead to more sustainable performance (Barney et al., 2001). Thirdly, due to its flexibility, using the TOE framework will allow the incorporation of many technological, organisational and environmental factors (Chege & Wang, 2020a), moreover, the TOE framework covers the wider context in which technology is used in SMEs (Chege & Wang, 2020b).

The COVID-19 crisis has affected the world economy in terms of international trade and tourism (Kuckertz et al., 2020). The negative impact spans across all spheres of human life, particularly with regards to health (Sigala, 2020). Most economic activities remain limited due to lockdowns, social distancing, travel restrictions and so on which is a substantial blow to many businesses (Brown & Rocha, 2020). Businesses and industries of all sizes have been severely impacted due to COVID-19 and are struggling to remain sustainable. However, some businesses have taken this opportunity to find a new niche for themselves, and many SMEs are trying to cope with this changed environment (Bretas & Alon, 2020). Due to their limited resources, SMEs can be more vulnerable to global crises, and COVID-19 is having a particularly harmful effect on them (Utomo et al., 2021). With sustainability becoming one of the most integral factors in the current environment, there remains a need to adopt operations to achieve long-term survival (Mustafa & Abbas, 2021). This situation pressurises SMEs to improve by employing technologies in order to operate sustainably (Winarsih et al., 2021). The technologies used to develop or adopt green practices lead to sustainability in the industry (Mustafa & Abbas, 2021). The current research will focus on the mechanism of sustainable performance that SMEs can adopt during the COVID-19 pandemic.

Limited empirical research exists on the effect of technological innovation on sustainable performance through adoption of sustainable green practices in the SME sector (Chege & Wang, 2020a; D. Singh, Khamba, et al., 2017; Singh, Tan, et al., 2017). To fill this gap, further exploration is required. The average of organisations making use of technology is usually 2 percent (Chege et al., 2020). However, the current COVID-19 pandemic has made it inevitable that organisations will shift from traditional to green practices to perform sustainably. For this purpose, SMEs will require customised technologies. The available research on SMEs covers the determinants of the implementation of environmental and social practices, including environment productivity and performance (Rahman & Post, 2012; Revell et al., 2009), environmental and social practices (Chang et al., 2018), social performance (Sutantoputra, 2009) and green innovation (Arnold, 2017). However, they do not cover the influence of technological innovation on sustainable practices and their effect on SMEs’ sustainable performance. The context is generally Western, Asian or African. The specific Middle Eastern region is generally neglected. The current research aims to contribute to both theory and practice by developing a model expected to provide an inclusive theoretical framework for measuring how technological innovation can lead SMEs’ sustainable performance levels through adoption of sustainable green practices.

## Study Context

The Sultanate of Oman is one of the prominent members of GCC with a rural and agrarian economy. Oman’s population is around 4.56 million with 54.30% Omani nationals compared to 45.70% expatriates according to the National Centre for Statistics and Information, 2017 (Imran & Al-Ansi, 2019). Moreover, the latest data from world bank showed that the Omani GDP reached to 76.33 billion US dollars in 2019 while the GDP value of Oman represents 0.07% of the world economy (TE, 2021). Oman is characterized by its consistent, systematic and impressive development. Traditionally, its economy has predominantly relied on oil revenues, but recent fluctuations in oil demand and prices have contributed to the use of other sources of income, seeking for more sustainable development (Alraja et al., 2021; Magd & McCoy, 2014). In addition, the Covid-19 pandemic has resulted in many negative economic impacts worldwide. The Omani economy has also been significantlyaffected, leaving many SMEs suffering. But, according to the International Monetary, the Omani economy is expected to recover in 2021 with a projected growth of 2.5 percent after a 2.8 percent contraction in 2020 with 1.5% growth from non-oil activities compared to a 3.9% contraction in 2020 (Kamel, 2021). Furthermore, the pressure on the economy was one of key factors influencing Omani business interests towards sustainable goals. A number of institutions have been established to invest towards a sustainable green economy, including research centers, Sultan Qaboos University, the Innovation and Development Center, Business Incubators and the Scientific Research Council. Moreover, the Environment Society of Oman was established in 2004, which aims to increase environmental awareness and capacity building (Siyabi & Hakro, 2020). Pressure on governmental resources has reduced, as many initiatives have started materialise, such as technological advancements, promoting private sector investments and encouraging entrepreneurial startups and SMEs (Magd & El-Gharib, 2021). The country has a large geographical area as compared to its population thus the basic facilities and the technological advancements are required for inter-connectivity in the economy (Alraja et al., 2021). The COVID-19 crisis has further pushed Omani organizations, especially SMEs, to find sustainable ways of conducting business, however the absence of relevant research is hampering progress. This research aims to help bridge this gap and is among the pioneer projects focused on the mechanism of sustainable performance that SMEs can adopt.

### Theoretical Framework and Hypotheses Development

The adoption of technological innovations has undergone investigation using different models and theories such as economic theory (Omri, 2020) and open systems theory (Cancino et al., 2018). This study has adopted the Technology-Organisation-Environment (TOE) model developed by Tornatzky and Fleischer (1990) as the primary theoretical underpinning. This model allows organisations to integrate the wider use of technology (Chege & Wang, 2020a). This framework suggests that the capabilities of the firm to adopt and implement technological innovation depends on three main factors in organizations, namely technological factors, organisational factors and environmental factors. The technological factors in SMEs can be internal and external, for instance, their internal IT infrastructure or the wider external communication infrastructure such as suitable Internet access etc., while the organizational factors can be described as internal, such as management support, size of the firm and ICT innovation intensity, whereas the environmental factors are external such as government regulations, green technologies support infrastructure and pressure from consumer and environment campaigners (Chege & Wang, 2020a).

**Fig. 1:**
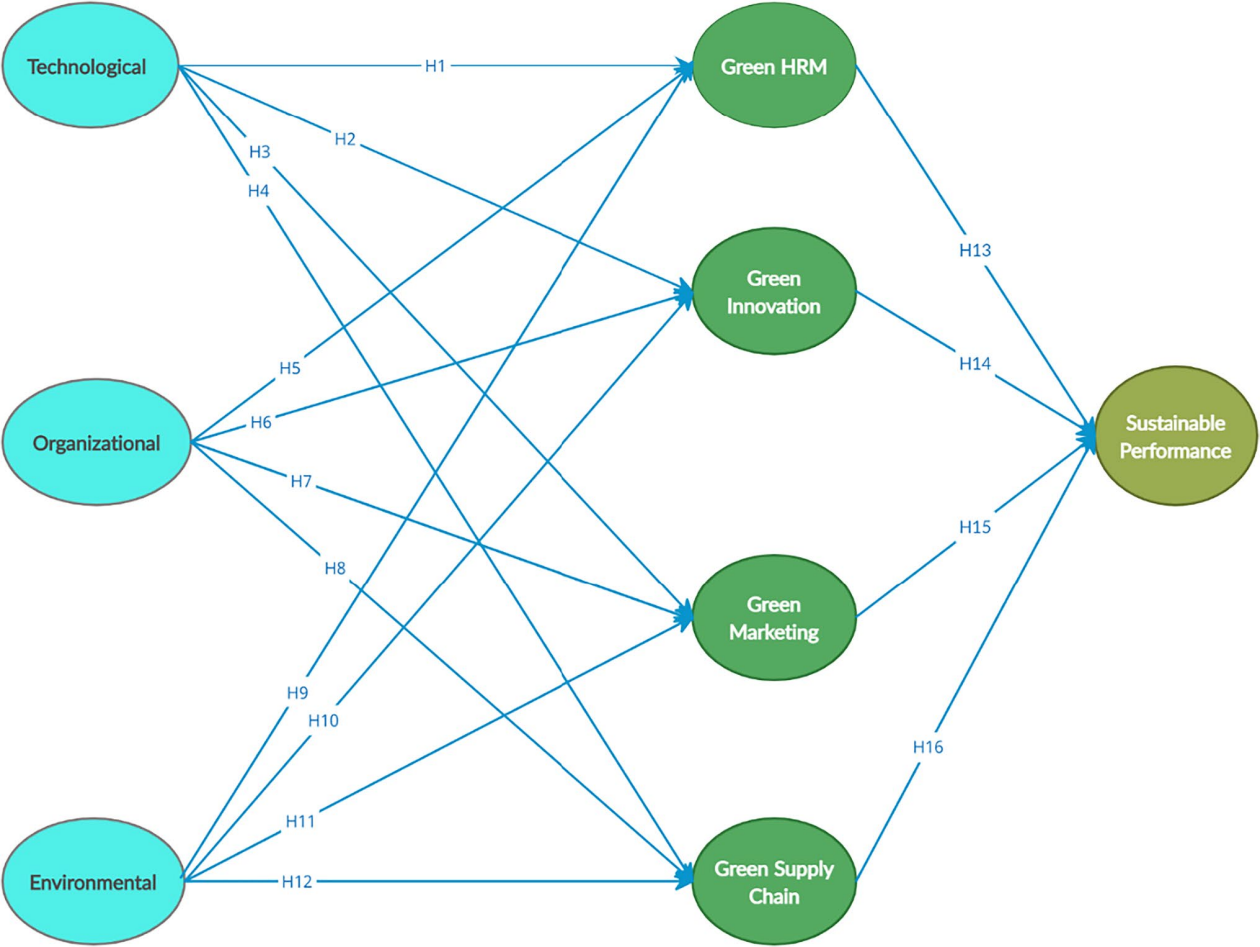
Proposed model

Technological innovations are an integral source of sustainable green transformations (Effendi et al., 2020; Yahya et al., 2014). The improvements in technological innovations have led to waste reduction and sustainable green practices (Wang et al., 2021). The sustainable green practices identified in the literature are different and include the green supply chain (Centobelli et al., 2020), green HRM (Mousa & Othman, 2020), Green marketing (Chung, 2020) and green innovation (Asadi et al., 2020). The current study included a combination (represented in Fig. 1) of these sustainable green practices, including green HRM, green innovation, green marketing and supply chain management.

### Technological Factors and Sustainable Green Practices

The technological factors refer to the relevant internal and external technologies for the organization that fits its existing technology landscape (Tornatzky et al., 1990). The frequently used constructs are relative advantage, complexity and compatibility (Grover, 1993; Ramdani et al., 2009). Relative advantage is the advantage gained from the innovation as compared to the one it replaces (Sahin, 2006). Complexity on the other hand, is the perceived difficulty in developing, implementing or utilizing an innovation (Sahin, 2006). Comparability is the consistency of the innovation with the existing worth, experiences and customer needs (Sahin, 2006).

Technological factors improve technological innovation. One of the practices of technological innovation involves adopting a system for environmental management (Lin & Ho, 2011). There are numerous factors affecting technological innovation (Chege & Wang, 2020a; S. Singh, Khamba, et al., 2017; Singh, Tan, et al., 2017). However, the current research focuses on the factors of relative advantage and complexity that bear a positive association with maintaining the environmental impact or by the adoption of environmental management systems (Effendi et al., 2020; Lin & Ho, 2011; Singh Khamba, & Nanda, 2017; Singh, Tan, et al., 2017). Relative advantage represents one of the technological factors that describes the perceived benefit of the innovation, with organisations tending to adopt technology that demonstrates better performance and high economic gains (Rogers, 2010; Tornatzky & Klein, 1982). The relative advantage of technological innovations influences innovation adoption and has a positive relationship with the adoption of sustainable green practices (Lin & Ho, 2011; Singh et al., 2017a, 2017b). Compatibility, on the other hand, represents the perceived consistency of the innovation with firms’ prevailing requirements, standards and practices (Everett M. Rogers, 2003). It is relevant to sustainable green practices because the alignment between technological innovations’ with existing technologies simplifies the diffusion or adoption of green practices (Etzion, 2007; Lin & Ho, 2011).

The technological factors simplify the adoption of sustainable green practices (Thomas et al., 2016). One of the most integral sustainable practices is green HRM. People management takes its inspiration from significant technological advancements. Technological factors are related to the equipment, software and hardware and the ability to introduce new systems (Armstrong & Taylor, 2017; Yerkes, 2003). The presence of relevant and compatible technological factors not only increases the speed and efficiency of the process but often also minimize the cost and improve the productivity (Marler & Fisher, 2013). It also eases the process for firms to develop and adopt green HR practices (Rahman et al., 2018; Waheed et al., 2019). The technological factors of the TOE framework relate to the development of organizational infrastructure such as hardware, software and technological equipment (Rahman & Aydin, 2019; Rahman et al., 2018). Moreover, once the organizations introduce innovative technologies it challenges the status quo and has an effect on organizational operations (Armstrong, 2016). Technological factors also have a strong influence on the adoption of a green supply chain. Such chains facilitate the performance of supply chain practices and improve the coordination and physical flow of information within the system (Omar et al., 2010). The adoption of a green supply chain is a complex process that requires consideration of many aspects (Hsu & Hu, 2008). The technological factor of the TOE framework demands that organizations to adopt innovations and technologies related to better performance and higher economic gains (Hanna et al., 2021), i.e. how much these technologies are consistent with the present structure and requirements (Rogers, 2010). The fit between the existing system and relevant technologies leads towards the adoption of the green supply chain (Hwang et al., 2016; Savita et al., 2016; Yang et al., 2018).

The rise in the requirement of green concepts and the use of relevant technological factors can also affect green marketing strategies (Chung, 2020). The competition has forced the organizations to adopt green marketing practices (Kumar, 2015). The presence of technological factors makes the adoption of green marketing practices easy in order to meet sustainability goals (Karjaluoto & Vaccaro, 2009). Organisations’ preparations in terms of the availability of technological factors make them capable of innovating and controlling potential dangers (Jones et al., 2005). Thus, the relevant and compatible technological factors facilitate the adoption of green innovation (Chong & Olesen, 2017; Zhang et al., 2020). The adoption of green innovation practices is largely dependent on the technological factors. The value of the technological factors is dependent on their compatibility with the other technologies needed for green innovations (Zhang et al., 2020). Evidence suggests that the SMEs should make use of technological factors and adopt sustainable green practices, especially during the COVID-19 pandemic (Chege & Wang, 2020a; Omri, 2020; Ryoo & Koo, 2013). Therefore, the following hypotheses are proposed:

- Technological factors have a positive effect on adoption of (H_1_) green HRM (H_2_) green innovation (H_3_) green marketing (H_4_) green supply chain.

### Organisational Factors and Sustainable Green Practices

In the research on technological innovation, organisational factors account for the second component in the TOE model. Many constructs have been used to define the organisational factors (Chege & Wang, 2020a; S. Singh, Khamba, et al., 2017; Singh, Tan, et al., 2017). The characteristics of organizational resources including firm size, communication process and management support (Baker, 2012). Some of the important constructs are firm size, top management support and IT skills of non-IT employees (Borgman et al., 2013). In adoption of technological innovations, the most important factor is the size of the organization. Large organizations usually are in a better position to not only invest in innovations but also to facilitate its implementation (Sahin, 2006; Thong, 2015). Top management support is necessary to create a conducive environment and ensure resource provision for technological innovations (Chau & Jim, 2002; Grover, 1993; Ramdani et al., 2009). The organizational factors of technological innovations are also impacted by the IT skills of non-IT employees; it is the crucial factor in this regard (Chau & Jim, 2002; Grover, 1993). These variables have a positive association with the adoption of environmental management systems (Effendi et al., 2020; Lin & Ho, 2011; Singh Khamba, & Nanda, 2017; Singh, Tan, et al., 2017).

The organizational factors of technological innovations and the adoption of green practices constitute complex processes that require high-level training and human resources development (Del Brío & Junquera, 2003; Hart, 1995). The efficacy of training programmes depends on the competence and capability of the employees (Russo & Fouts, 1997). Therefore, the quality of human resources has a positive effect on the adoption of sustainable green practices (Lin & Ho, 2011; Singh, Khamba, et al., 2017; Singh, Tan, et al., 2017). Adoption of sustainable green practices also depends on the extent of organisational support. Motivated employees demonstrate a greater likelihood to adopt green practices and implement green behaviours. Thus, the presence of organisational support makes the adoption of green practices easy (Lin & Ho, 2011).

The adoption of sustainable green practices becomes smoother due to the presence of relevant factors. Moreover, the adoption of green HRM requires relevant factors, as HRM symbolises the integral aspect of the organisation. The presence of required organisational factors facilitates green HR practices (Rahman et al., 2018; Waheed et al., 2019). The organizational context of the TOE framework refers to the human assets and relevant characteristics such as organizational structure, size and process of communication (Rosli et al., 2012). For implementing green HRM, these factors play an important role (Rahman et al., 2018; Ruël et al., 2004). An organisation’s choice to adopt green supply chain practices also necessitates the presence of relevant organisational factors. The presence of qualified human resources and relevant support facilitates the adoption of green supply chain practices (Hwang et al., 2016; Savita et al., 2016). The organisations differ in their internal resources, procedures and ability to respond to challenges. The organizational factors that are unique to an organization can actually build competitive advantage. To adopt green supply chain management practices, the organizational factors play an important role (Hwang et al., 2016). Marketing represents an integral factor in organisational success. Adoption of green marketing practices can enhance sustainability. However, the adoption of green marketing practices requires supportive organisational factors (Chung, 2020). The presence of relevant and supportive organizational factors according to the TOE framework makes it easier for organizations to adopt green marketing practices (Kumar, 2015). Organizational motivation is essential for green innovations (Tsai & Liao, 2017). The presence of organizational factors necessary for green innovation facilitates its adoption (Aboelmaged & Hashem, 2019). Organisations competing in an external environment rely on green products and process innovation as a source of competitive advantage. However, it is largely dependent on internal factors. The adoption and implementation of green innovations rely upon the quality of organizational factors (Chong & Olesen, 2017; Zhang et al., 2020). Therefore, the following hypotheses are proposed:

- Organisational factors have a positive effect on adoption of (H_5_) green HRM (H_6_) green innovation (H_7_) green marketing (H_8_) green supply chain

### Environmental Factors and Sustainable Green Practices

The environmental factors component of the TOE model indicates the external environment of the organisation. SMEs’ willingness to adopt sustainable green practices largely depends upon government support (Lee, 2008). In an uncertain environment, technological innovation will improve organisations’ capabilities to adopt sustainable green practices (Aragón-Correa & Sharma, 2003; Rothenberg & Zyglidopoulos, 2007).

Environmental factors have a significant impact on the adoption of sustainable green practices. Green human resource management practices cannot be adopted without external support. The organisation committed to adopting green practices may fail in the absence of government support. Therefore, the support of environmental factors facilitates the adoption of green HR practices (Rahman et al., 2018; Waheed et al., 2019). The environmental context of the TOE refers to external environmental issues which are a major push towards adoption of green HRM (Rahman & Aydin, 2019; Rahman et al., 2018). Supply chain management comprises external activities. Thus, the kind of factors around the organisation can affect the type of supply chain practices adopted. Environmental regulations have emerged as a key driver to the adoption of sustainable green practices (Aboelmaged, 2018). An organisation’s decision to adopt green supply chain practices requires the presence of relevant environmental factors (Hwang et al., 2016; Savita et al., 2016). The environmental factors also influence the marketing practices. The relevant environmental factors actually facilitate the adoption of green marketing practices and green marketing practices only prove successful in a supportive external environment. The adoption of green marketing practices requires support from environmental factors (Chung, 2020; Kumar, 2015). The environmental factors push organizations towards adoption of green innovation. The government and legal environmental protection policies have an impact on an organization’s innovative practices (Chen et al., 2018; Huang et al., 2016). The process of adoption of green innovation largely depends on the prevailing environmental factors such as support, certainty, policy orientation and market orientation (Zhang et al., 2020). These factors can trigger the kind and type of innovations. Thus, the adoption of green innovations also depends on environmental factors (Chong & Olesen, 2017; Zhang et al., 2020). Therefore, the following hypotheses are proposed:

- Environmental factors have a positive effect on adoption of (H_9_) green HRM (H_10_) green innovation (H_11_) green marketing (H_12_) green supply chain.

### Sustainable Green Practices and Sustainable Performance

Sustainability represents one of the most pressing issues in the current climate (Al Hammadi & Hussain, 2019). The emerging environmental issues constitute a trigger for organisations to find alternative means to survive (Saudi et al., 2019). To succeed in the dynamic business environment, SMEs face overwhelming challenges to implementing sustainable practices (Chang et al., 2018). Their task is to use their limited resources and implement sustainable practices to achieve long-term sustainability (Chege & Wang, 2020a; Omri, 2020). The resource-based view proposes that the distinctive capabilities and sustainable practices of the organisations result in a competitive advantage and sustainable performance levels (Alshehhi et al., 2018).

Past literature, however, is limited in examining the effect between sustainable practices and sustainable performance. Moreover, the literature has taken different sustainable practices, such as environmental practices and green practices, linked with sustainable performance (Borga et al., 2009; Phan et al., 2020; Reyes-Rodríguez et al., 2016). The COVID-19 pandemic completely altered the operating environment. This situation provides the foundation to probe the relationship in detail in this specific situation. Past literature has examined the individual green practices or a combination of two practices, yet a holistic model is absent. The current pandemic has adversely hit organisations, especially SMEs (Block et al., 2021; Fitriasari, 2020) and has highlighted the importance of sustainable performance. Thus, the adoption of sustainable practices represents the only solution to achieve sustainable performance.

Environmental issues are emerging as managerial challenges as organisations struggle to identify ways to reduce negative environmental impact and achieve sustainable performance (Ahmad, 2015; Mancha & Yoder, 2015). Organisations adopt different sustainable green practices, such as green HRM, to cope with environmental issues and achieve sustainable performance. Green HRM comprises the environmental practices that make organisations more sustainable. These practices ensure sustainable performance (Khan et al., 2021; Mousa & Othman, 2020). Organisations can achieve sustainable performance through adopting green HRM practice (El-Kassar & Singh, 2019; Mousa & Othman, 2020). The emerging environmental issues have forced organisations to shift from traditional to green innovations to survive (Khan et al., 2018; Mohd Saudi et al., 2019).

Enhancing green innovation for corporate sustainability is one of the recent issues globally (Shahzad et al., 2020). Adoption of green innovation practices is interconnected with an organization’s environmental schema (Adegbile et al., 2017). Green innovation in both processes and products have a positive effect on not only the environment but also cost reduction and sustainable performance (Singh et al., 2020). Literature suggest that adoption of green innovation practice is an organization’s intention towards environmental issues and achieving long-term performance (Kratzer et al., 2017; Lin et al., 2013). The resource-based view also suggests that green innovation can aid competitive advantage and result in sustainable performance (Singh et al., 2020).

One of the ways to ensure sustainable production and consumption and ultimately sustainable development is for businesses to have sustainable or green marketing practices (Fatoki, 2019). Green marketing practices lead to sustainability (Papadas et al., 2017). The adoption of these practices meets environmental concerns. Such practices address environmental challenges and share a link with sustainable performance (Kinoti, 2011). The concept of green marketing has been conceptualized and tested with performance and sustainability in the past (Hasan & Ali, 2015; Kinoti, 2011; Lam & Li, 2019) however, there is a need for further in depth exploration.

Nowadays, organizations adopt green supply chain management for addressing the needs of the community and stakeholders. The organisations, in order to sustain themselves, should adopt environmentally friendly green supply chain practices and reach their performance-related outcomes. Green supply chain management practices help organizations to attain their economic and environmental goals and achieve sustainability (Das, 2018). The firms with green supply chain objectives always focus on their suppliers to be green and follow green practices to meet their performance-related objectives (Heras-Saizarbitoria et al., 2011).

The concept of green supply chain management and its relationship with performance has been the center of attention in the past few years (Bon, 2018; Das, 2018; Mitra & Datta, 2014). The firm-level strategic resources, such as the green supply chain, result in sustainable performance (Fatoki, 2019).

Thus, following hypotheses are proposed:

- H_13_: green sustainable practices (H_14_) green innovation (H_15_) green marketing (H_16_) green supply chain adoption has a positive effect on sustainable performance.

## Methodology

### Sample and Data Collection

SMEs play a vital role in the economy (Alraja et al., 2021), especially in achieving sustainability, as these enterprises have an integral effect on many sustainable development goals (Betti et al., 2018). The research focused on SMEs from different sectors in Oman. Table 1 illustrates the profile of the targeted organisations.

**Table 1:** Enterprises profile

Characterization		Frequency	Percent	
SME Size	5 -9 employees	251		37.5
	10—19 employees	157		23.5
	20—49 employees	201		30.0
	50—99 employees	60		9.0
SME Age	less than 5	351		52.5
	5 to less than 10	114		17.0
	10 to less than 15	64		9.6
	15 to less than 20	69		10.3
	20 and more	71		10.6

SMEs in Oman are active in all different industries i.e. manufacturing and service industry. SMEs are expected to play a vital role in the economy. During the pandemic, SMEs helped both business and individuals reduce the negative effect on them i.e. SMEs were able to make the vital material and goods available for people, shops and other business. For example, during COVID-19, SMEs focused digital solutions as in person contact was prohibited (Times of Oman, 2021). Compared with 2020, the number of operating SMEs in Oman has increased from 44,139 to 52,524 SMEs (+ 7000) (NCSI, 2021). However, our research focused on SMEs from different sectors in Oman and different areas of Oman; especially from Muscat, which is home to 17,692 SMEs (the largest in Oman).

We chose a broad sample of SMEs to get input from a wide range of organisations and people working in those. For example, in our sample, 52.5% were less than 5 years old, 17% were 5 –10 years old, 9.6% were 10 –15 years old, 10.3% were 10 –15 years old and 10.6% were 20 years old and more. Medium sized enterprises (10—99 workers) made up 63.5% and small sized enterprises (5—9 workers) made up 37.5%. Furthermore, about 70% of the respondents are female, while 30% were male. It has been reported that Omani’s total early-stage entrepreneurial activity rate has more than doubled between 2019 and 2020 with a significant increase from 6.7% to 16% explaining the increased desire of Omani adults to start a new business in comparison to previous years (GEM, 2020). Numerous studies conducted in developing countries like Oman (Ghouse et al., 2017) found that a female’s entrepreneurial skills can be key drivers of the social and economic development (Ghouse et al., 2019); most Omani SMEs are found to be operated by women (GEM, 2005). In 2021, a report by GEM showed that the majority of new Omani firms are regularly expected to be started by female entrepreneurs as the rates have increased by at least 10% compared to men (Bosma et al., 2021).

Furthermore, about 23% of the employees were from international backgrounds, and 4.3% of the targeted SMEs were international enterprises. However, the employees in our sample belong to different units and levels in their SMEs. i.e. about twenty five percent belong to a marketing unit/department, 23.8% to a human resources unit/department, 19.6% to an accounting/finance unit/department, 16.4% to an IT a unit/department, 11.2% to a customer services unit/department and 3.6% to a other unit/department (refer to Table 2).

**Table 2:** Respondents profile

Characterization		Frequency	Percent		
Gender	Male	208			31.1
	Female	461			68.9
Employee type (national or international)	National	517			77.3
	International	152			22.7
Employees work in Local or International enterprise	Local company	640			95.7
	International enterprise	29			4.3
Specialization	Marketing unit/department	170			25.4
	Human Resources unit/department	159			23.8
	Accounting/finance unit/department	131			19.6
	IT unit/department	110			16.4
	Customer services unit/department	75			11.2
	Other unit/department	24			3.6
Job level	First Line Employee	286			42.8
	First Line Manager	133	19.9		
	Middle Level Manager	169		25.3	
	Top Manager	81		12.1	

The definition of SMEs tends to rely on the number of employees, meaning the researchers grouped the surveyed SMEs into four primary groups to meet the majority definitions. In Oman, small enterprises have between five and nine employees, while medium enterprises employ between 10 and 99 workers (MTC&IT 2021).

However, the online administered questionnaire was available to respondents in both English and Arabic languages. A purposive sampling procedure took place to select the sample i.e. based on the available list of SMEs, the electronic link of our questionnaire was submitted to all SMEs that have an available email or any other electronic communication method like WhatsApp (this method was adopted to avoid any physical contact between people and maintain social distancing to reduce COVID-19 spread). An introductory paragraph articulated the purpose of the study and defined key terms. Following the Oman definition of SMEs (MTC&IT 2021), all Omani SMEs that met the identified criteria were targeted purposively. Furthermore, all the employees who belong to one of the following groups 'First Line Employee', 'First Line Manager', 'Middle Level Manager' and 'Top Manager’ were encouraged to respond to the online distributed questionnaire. The research model was tested using data collected between March and April 2021.

### Common Method Bias

The respondents in this study came from different administrative levels and functional specialisations (see Table 2 respondents’ profile) working in Oman’s SMEs. However, to avoid any potential common method bias (CMB), the study followed the Ping's (2004) suggested steps before, during and after data collection. To judge consistency, validity and reliability prior to data collection, **firstly**, a group of experts comprising five academicians and five experts from the SMEs sector reviewed the developed document. **Secondly**, based on the comments and feedback, the first draft of the questionnaire was modified, with a pilot study then taking place on a purposive sample of 30 employees from SMEs to ensure the validity and reliability of the questionnaire before its distribution for data collection (van Teijlingen & Hundley, 2002). Subsequently, the final version of the questionnaire was made ready. Afterwards, during data collection, targeted participants received a briefing about the study’s aims through a cover letter. This method ensured the participants that their responses would remain anonymous and confidential and would only be used for academic research. Additionally, it encouraged them to answer questions truthfully and reminded them that they could withdraw from the survey at any point. Finally, the study constructs were separated randomly in the final version of the distributed questionnaire. After data collection, researchers implemented Harman's single-factor test to check the presence of CMB. The test showed seven factors, and the highest variance for the first rotated factor was 40.786 percent (Podsakoff et al., 2012). This variance stood at less than the accepted 50 percent threshold, which indicates no pressing issues related to CMB in the current study (Pinzone et al., 2019).

### Measures

This study assessed the connection shared by technological innovation (as inputs) represented by technological, organisational and environmental factors, green practices (as process) represented by green human resources management, green marketing, green innovation and green supply chain, and sustainable performance (as outputs). The study also examined the causal impact of technological innovation on green practices, as well as the impact of green practices on sustainable performance. To examine this conceptual model, a survey instrument was designed using items from some previously developed instruments. This survey underwent translation into Arabic to ensure clarity for participants. The translated version was reviewed by two bilingual faculty members belonging to the same field of the current study to aid understanding for respondents unfamiliar with the English language, thereby helping them select items that reflected their opinion accurately (Alraja et al., 2019, 2020). The measurement was perception-based, and the unit of analysis was individual, with all scales having multiple dimensions. **Technological innovation** was measured through 18 items divided into three dimensions of technological factors, organisational factor and environmental factors which were measured by seven, six and five items, respectively. These factors were validated by previous research and adopted from (Chege & Wang, 2020a). Meanwhile, green practices were measured through 22 items divided into four dimensions of green HRM, green marketing, green innovation and green supply chain. **Green HRM** was evaluated with six items utilised from (Guerci et al., 2016). Moreover, **green innovation** was assessed using six items adopted from (Aboelmaged & Hashem, 2019; Chen & Liu, 2020; Chiou et al., 2011), **green marketing** was tested using five items adopted from [80], and **green supply chain** was examined using five items adopted from (Benzidia et al., 2021; Chiou et al., 2011; Singh & El-Kassar, 2019). **Sustainable performance** was measured through five items adopted from (Lin et al., 2013).

### Statistical Analysis Procedure

The questionnaire was administered online. All the received responses underwent initial scanning using SPSS 23 software. The process brought in 756 questionnaires. After assessing the returned responses and based on the initial scanning, 87 were dropped from the analysis because the questionnaire items had given the same answer (e.g. all questions were strongly agreed or disagreed), and in some of them, the size of the enterprise was out of the acceptable range (i.e., more than 4 employees and less than 100). Consequently, the valid questionnaires for analysis were 669, which accounted for 88.5 percent of completed questionnaires. This sample size fell within the acceptable range for analysis using partial least squares (PLS) (Hair et al., 2016). The structural model of the current study underwent assessment through the partial least squares-structural equation modelling (PLS-SEM) (Ameen, et al., 2021a, 2021b; Henseler et al., 2009) using the SmartPLS 3.3.3 software.

The final valid data (questionnaires) underwent analysis in two primary phases. The first phases sought to validate the adopted measurement through testing. The skewness and kurtosis test ensured the normal distribution of the utilised items while the composite reliability (CR) and Cronbach's alpha (**α**) tests ensured the internal consistency reliability of the study mode. Then, convergent and discriminant validity tests validated the research model. Prior to carrying out the structural equation modelling test, the multicollinearity test (inner and outer test) took place using the variance inflation factor (VIF) method to determine whether any potential errors could arise due to the high correlations of the latent variables.

## Analysis and Results

### Measurement Assessment

The validity of the measurement model underwent appraisal using different types of tests, and the normal distribution was ensured by the statistical tests skewness and kurtosis, with the result for each item falling in the acceptable range + 2 to -2.

Moreover, the outer loading (represented in Table 3) ensures that loadings of all included items exceeded the acceptable threshold ≥ 0.70 (Hair et al., 2010a). However, the loading value for a few items was < 0.70, so they were dropped from the analysis. Such items included the technological latent variable: indicators Tech8 (0.639), and Tech9 (0.612); the green innovation latent variable: indicators GI1 (0.691) and GI3 (0.692); the organisational latent variable: item Orga7 (0.635); and the sustainable performance variable: indicator SP1 (0.627). After these eradications from the dataset, the data underwent analysis again, resulting in the indicator reliability of > 0.70. Appendix 1 shows all measurement items, including those deleted due to the low loading value. Furthermore, all latent variables achieved the cut-off value of > 0.70 (Hair et al., 2019) for both composite reliability (CR) and Cronbach’s alpha (α).

**Table 3:** Measurement assessment results

Variable	Item	Alpha	CR	AVE	Loadings
SP	SP2	0.843	0.889	0.615	0.778
	SP3				0.755
	SP4				0.812
	SP5				0.787
	SP6				0.787
GHRM	GHRM1	0.877	0.907	0.620	0.769
	GHRM2				0.773
	GHRM3				0.821
	GHRM4				0.816
	GHRM5				0.797
	GHRM6				0.744
GI	GI2	0.868	0.901	0.603	0.762
	GI4				0.750
	GI5				0.752
	GI6				0.825
	GI7				0.778
	GI8				0.790
Tech	Tech1	0.879	0.906	0.581	0.747
	Tech2				0.757
	Tech3				0.804
	Tech4				0.796
	Tech5				0.785
	Tech6				0.725
	Tech7				0.717
Orga	Orga1	0.879	0.908	0.642	0.749
	Orga2				0.822
	Orga3				0.799
	Orga4				0.818
	Orga5				0.806
	Orga6				0.740
Envi	Envi1	0.866	0.903	0.651	0.796
	Envi2				0.787
	Envi3				0.842
	Envi4				0.827
	Envi5				0.782
GSC	GSC1	0.861	0.900	0.642	0.780
	GSC2				0.818
	GSC3				0.810
	GSC4				0.811
	GSC5				0.787
GM	GM1	0.828	0.879	0.593	0.792
	GM2				0.769
	GM3				0.809
	GM4				0.771
	GM5				0.707

### Model Validity

The study model validity was ensured by the convergent validity. The calculated values of the average variance extracted (AVE) for all constructs were > 0.50 (Fornell & Larcker, 1981; Hair et al., 2016), (see Table 3), which confirmed convergent validity. Moreover, discriminant validity underwent testing using the following methods:

Firstly, the Fornell-Larcker criterion, as shown in Table 6 (Appendix 2), indicated that the square root of the AVE for each latent variable proved higher than its maximum correlation with other latent variables (Fornell & Larcker, 1981).

Secondly, the cross-loadings matrix, represented in Table 7 (Appendix 2), showed each indicator the outer loading on its related latent variable proved greater than all its cross-loadings with other latent variables (Hair et al., 2019).

Thirdly, the use of the heterotrait-monotrait (HTMT) ratio (Henseler et al., 2015), compensated for the lack of sensitivity of the Fornell-Larcker criterion and cross-loading methods to document discriminant validity. For all adopted variables, the optimal HTMT values < 0.85 were achieved, as shown in Table 8 (Appendix 2). Hence, based on the above-conducted tests, the adopted model demonstrates the presence of discriminant validity.

### Analysis of Structural Model

Prior to the structural modelling test taking place, the variance inflation factor (VIF) method sought to exclude any potential errors that may originate from high correlations between the adopted latent variables (Hair et al., 2010b). With PLS-SEM, a collinearity issue was indicated by VIF ≥ 3.3 (Ameen, et al., 2021a, 2021b; Petter et al., 2007). Table 9 (Appendix 2) depict all VIF (outer and inner) values below this threshold. Thus, in addition to the results of Harman’s single factor test discussed previously, both results ensure the absence of multicollinearity problems.

**Fig. 2:**
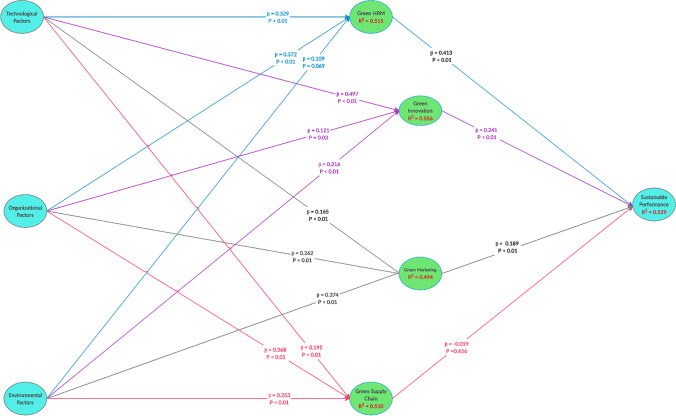
Structural model path coefficients

Figure 2 shows all path coefficients (β) values from the model’s construct relationships. A bootstrapping algorithm with 5000 bootstrap samples in PLS was implemented to determine significance (Table 4). Five percent error probability for the (β) values was appraised from t and p values, i.e., p ≤ 0.05 validated the hypothesis; t value > 1.96.

**Table 4:** Hypotheses test results

Hypothesis	Path	β coefficients	T Statistics	P Values	Result
H1	Organizational → Green HRM	0.37	6.57	0.00	Support
H2	Organizational → Green Innovation	0.12	2.15	0.03	Support
H3	Organizational → Green marketing	0.26	5.29	0.00	Support
H4	Organizational → Green supply chain	0.37	4.72	0.00	Support
H5	Technological → Green HRM	0.33	5.86	0.00	Support
H6	Technological → Green Innovation	0.50	9.07	0.00	Support
H7	Technological → Green marketing	0.17	2.98	0.00	Support
H8	Technological → Green supply chain	0.20	2.91	0.00	Support
H9	Environmental → Green HRM	0.11	1.85	0.07	Reject
H10	Environmental → Green Innovation	0.22	4.22	0.00	Support
H11	Environmental → Green marketing	0.37	8.67	0.00	Support
H12	Environmental → Green supply chain	0.25	3.33	0.00	Support
H13	Green HRM → sustainable performance	0.41	7.89	0.00	Support
H14	Green Innovation → sustainable performance	0.24	4.26	0.00	Support
H15	Green marketing → sustainable performance	0.19	4.24	0.00	Support
H16	Green supply chain → sustainable performance	-0.02	0.45	0.66	Reject

Organisational factors increase green HRM, green innovation, green marketing and green supply chain (β = 0.37, 0.12, 0.26 & 0.37 respectively). Further, technological factors also enhance green HRM, green innovation, green marketing and green supply chain (β = 0.33, 0.50, 0.17 & 0.20 respectively). Furthermore, environmental factors increase green innovation, green marketing and green supply chain (β = 0.22, 0.37, 0.25 & 0.41 respectively). Moreover, green HRM, green innovation, and green marketing improve sustainable performance (β = 0.41, 0.24, & 0.19 respectively).

On the other hand, environmental factors demonstrated no significant effect on green human resources management (β = 0.109; p > 0.05). Additionally, the green supply chain shows no significant effect on sustainable performance (β = -0.019; p > 0.05), meaning the rejection of both H9 and H16. Meanwhile, the path coefficients (β) values and p values for all remaining constructs were significant. Hence, all the remaining hypotheses were supported. Further, the dependent latent variable (sustainable performance) relative to the total variance underwent assessment based on the coefficient of determination, R^2^. As shown in Fig. 2, about 51.5 percent, 55.6 percent, 49.4 percent and 51 percent of the variance in GHRM, GI, GM and GSC, respectively, was described by technological, organisational and environmental factors. Together, GHRM, GI, GM and GSC explained a 52.9 percent of the variance in sustainable performance.

## Discussion

SMEs experienced setbacks due to the COVID-19 restrictions and faced many challenges to remain sustainable within this fragile environment. This study aimed to measure the impact of SMEs’ technological innovation (i.e., technological, organisational and environmental factors) on sustainable performance through sustainable practices such as green HRM, green supply chain, green innovation and green marketing.

This study represents one of the few pieces of pioneering research conducted during the COVID-19 pandemic that developed a holistic framework of sustainable performance, by combining the Technology-Organisation-Environment (TOE) framework and theory of resource-based view (RBV) as the theoretical underpinning. This integration sought to measure SMEs’ sustainable performance through green practices in the Middle Eastern region. Our results have shown the importance of environmental factors for green marketing as it has the highest impact level compared to other green practices.

These results are similar to recent study conducted by Chung (2020) indicating that green marketing plays key role in making businesses more competitive by considering environmental factors as drivers for the firm’s competitive values as main strategic goals.

While the environmental factors have affected the green supply chain and green innovation, they have shown no significant effect on green HRM. This situation implies that during the COVID-19 pandemic, managers of SMEs might have to decide between operating sustainably or folding as many other businesses did. Therefore, they focus less on the environmental factors concerning HRM practices as this crisis has forced people to work remotely, so they minimise their consumption of resources (raw materials, water and energy) and reduce emissions into the air. Thus, the demand from different environmental context pressures does not necessarily drive the SMEs to involve their employees in environmental issues or select their employees based on environmental criteria, especially under the disruption caused by COVID-19. Equally, minimising consumption of the company resources and its emissions into the air or water can influence the adoption of green marketing approaches and methods, such as encouraging the use of e-commerce, using more digital communication methods for promoting their products/services and applying a paperless policy in their procurement.

Although the study by Al-Sheyadi et al. (2019) supports the present belief that GSCM practices can meaningly improve the environmental performance of a business, but the pressure from a specific stakeholder group is one of the key drivers for emerging positive GSC practices. Accordingly, different performance outcomes can be related to the difference in contexts where GSCM practices are implemented. The Omani economy is growing fast where different international companies are operating. However, most of Omani SMEs are relatively smaller and newer i.e. less then 19 employees, and still at early stages of operating and investments i.e. less than 5. Although the Omani economy is growing fast where different international companies are operating, Omani laws and regulations regarding environmental management system (EMS) are still new, and therefore Omani SMEs might need to experience the long term outcomes before implementing GSC (Al-Sheyadi et al., 2019). This can justify why GSC does not improve performance as GSCM implementation practices require time, experienced leadership support and commitment. These findings are in line with Younis et al., (2019) who studied a similar context i.e. UAE and concluded that GSCM practices failed to influence environmental performance and eco-design & development (Deutz et al., 2013; Zhu & Sarkis, 2006). Another reason of the lack effect of GSC on sustainable performance is that according to RPT, it is possibly to state that supply chains under crisis are more likely to utilize their uncommon and unique internal resources wisely when framing strategies for achieving their competitive advantages, especially that current competition is more between supply chains comparing the individual companies (Geng et al., 2017). Companies could however share success stories on the benefits and positive effects of GSC practices on sustainable performance, thus encourage other business to adopt more ethical responsibility to protect the planet (Geng et al., 2017).

The majority of the physical/traditional marketing under this crisis has begun its transformation into digital processes. On other hand, in terms of internal factors, the organisational factors affected the green HRM and green supply chain, whereas the green innovation and green supply chain have, respectively, the highest impact on the technological factors. This finding aligns with (Hwang et al., 2016; Rahman et al., 2018). These results further support the idea that when Omani SMEs’ top management cares about their societal values and invest more time and effort in adoptung sustainable development practices, they are more likely to provide environmental training and advise their contractors and suppliers to follow the environmental criteria. This situation also accords with the earlier observations of (Hwang et al., 2016; Savita et al., 2016; Zhang et al., 2020).

This study’s findings suggest that investing in R & D to produce high-quality products or using new technology in the production process can help Omani SMEs use more environmentally friendly materials and cleaner or renewable technology to make more savings. This finding was also reported by (Chong & Olesen, 2017). These results also indicate that Omani SMEs, during the COVID-19 pandemic, use new technology to produce and deliver products and deal with responsible suppliers or subcontractors who adopt environmental criteria and engage in eco-friendly design and development. This finding broadly supports the work of other studies of (Hwang et al., 2016; Zhu et al., 2012) in linking technological factors with the green supply chain practice.

However, the data indicates that green HRM has been influenced by the internal factors (organisational and technological factors) but not the external factors (environmental). Such a situation can result from the internal influence of SMEs managers on organisational and technological factors to cope with the current pandemic. These observational findings suggest that attracting environmentally committed employees and providing environmental training to them is an indication that the Omani companies focus more on the organisational and technological factors.

These results are in agreement with those of (Mousa & Othman, 2020; Rahman et al., 2018; Waheed et al., 2019) who also found that technological and organizational factors are essential in facilitating the development process when adopting green HR practices.

Another integral finding was that green innovation has been impacted by all factors, with technological factors having the most pronounced effect. Such findings indicate that investing in new technology is essential for SMEs, especially under the current digital transformation culture, which seeks to improve the production process and quality. Prior studies that have noted the importance of technological factors for green innovation are many such as (Chong & Olesen, 2017; Zhang et al., 2020).

In terms of green marketing, all aspects of the TOE framework have a positive effect, with the external environmental factors having the largest impact while technological factors have the lowest. This situation demonstrates the significance of the imposed legal laws and other external stakeholders during the COVID-19 pandemic for SMEs to market their products/services in a more eco-friendly way using more online e-commerce platforms. This finding broadly supports the work of other studies such as (Chung, 2020 and Kumar, 2015) in this area linking environmental factors with green marketing.

However, the organisational factors have the highest effect on the green supply chain, while the environmental and technological factors show a medium impact. Rationally, the greater the adoption of sustainable development practices by Omani SMEs in times of crisis, the bigger the chance of advising and selecting suppliers or subcontractors that follow the required environmental criteria. This study supports evidence from previous observations (e.g. Hwang et al., 2016; Savita et al., 2016).

In terms of sustainable SME performance, the green HRM has shown the most positive development among all green practices, while no significant impact was evident between the green supply chain and sustainable SME performance. Moreover, green innovation and green marketing have a positive significance on sustainable SME performance. Hence, during COVID-19, the Omani SMEs that carefully appoint employees based on environmental criteria or/and through environmental commitment and provide training show greater likelihood to increase sustainability, such as by reducing hazardous waste/scrap, cutting the consumption of gasoline/fuel and building partnerships with green organisations and suppliers. In other words, SMEs’ performance sustainability is linked strongly with adopting HRM activities that encourage constructive and active environmentally friendly practices.

These findings may help understand the significance of green HRM practices, post-COVID-19, helping Omani SMEs avoid causing future harm by hiring employees who take responsibility for recycling and demonstrate commitment to the environment. These results corroborate the findings of a great deal of the previous work by (Aboelmaged & Hashem, 2019; J. Chen & Liu, 2020; Chiou et al., 2011; Muisyo & Qin, 2021). SMEs can be more sustainable if they improve their environmental compliance and use more environmentally friendly materials when redesigning and enhancing products or services that are easy to recycle, reuse and decompose, as found by (Papadas et al., 2017; Singh et al., 2020). Additionally, investing more in R & D to produce high-quality products and using new production and service delivery procedures will drive more SMEs to perform sustainably. These results align with other research such as (Chen, 2008; Chiou et al., 2011; Muisyo & Qin, 2021). However, adopting green supply chain activities are found not to contribute to the Omani SMEs’ sustainable performance. This result may be explained by the fact that travel restrictions and supply chain disturbances during the COVID-19 pandemic have influenced SMEs’ managers' decisions to select suppliers and subcontractors who can supply traditional materials or use more eco-friendly designs.

## Implications

### Theoretical Implications

This study developed a holistic framework of sustainable performance by combining a number of factors that showed a robust indication for achieving sustainable performance in SMEs. Integrating the model of the (TOE framework and the theory of RBV has provided a new perspective to measuring sustainability performance, especially through green practices as processes. In other words, the study has extended the TOE framework by adding a new green practices approach that has approved its validity as all TOE framework factors have demonstrated a positive coefficient when measuring the SMEs’ sustainability performances. Meanwhile, the TOE framework enables syndication of internal and external factors, whilst adding the green practices in alignment with the RBV has strengthened the theoretical view of sustainability performance. This study’s findings designate that green HRM, green innovation and green marketing play an imperative role in sustaining the Omani SMEs’ performance during the COVID-19 crisis. This combination provides strong support for the theoretical evidence that green practices as input have a crucial role in the sustainable performance of SMEs. However, green HRM should be the focus for Omani SMEs to sustain their performance. This study’s findings show the importance of SMEs’ organisational factors when adopting the green supply chain while succeeding in green marketing strategies requires Omani SMEs to focus more on environmental factors. However, as expected, the technological factors are the most essential variables for strategising the green innovation policies.

### Practical and Academic Implications

The results of this study inspire the Omani managers of SMEs and policymakers to focus more on the internal factors during the crisis, namely, technological and organisational factors, compared to the external environmental factors, and to encourage an eco-friendly culture in which different internal and external stakeholders adopt more environmental policies and regulations.

They can use this study’s findings to proactively develop sustainable performance strategies in response to the environmental rules and laws, especially during the pandemic. Moreover, this study’s findings suggest that variables such as green HRM, green marketing and green innovation directly affect sustainability. Therefore, during the pandemic, the managers and policymakers of Omani SMEs should 1) create roles including environmental responsibilities; 2) deliver environmental training to employees and managers; 3) recruit employees based on environmental criteria; 4) encourage the adoption of cleaner or renewable technology to make more savings; 5) focus on redesigning and improving products or services to meet new environmental criteria; 6) invest more in enhancing e-commerce and digital communication methods to promote their eco-friendly products/services; and 7) use recycled or reusable materials in their products/services. These criteria will enable the managers of SMEs to prioritise their internal resources for sustaining their performance.

Moreover, the absence of empirical evidence, and best practices relating to technological innovation, green practices and sustainable performance, will make this paper helpful for educational institutions. Teaching Omani SMEs about sustainability through green practices can help to boost the culture of green practice, especially in times of crisis where academic practical case studies can prove to be limited. Additionally, this study’s theoretical framework and the discussed findings can offer valuable knowledge for academic staff when developing curricula and teaching plans.

## Limitations and Future Research

This study has some limitations that could require additional research in future. Firstly, the developed model focused on the direct relationships of the constructs, meaning indirect investigations can help understand the impact of TOE factors on sustainable performance using green practices as moderators. Secondly, our study has used cross sectional data with mainly causality theoretical foundations; a longitudinal approach to future research could undergo investigation as TOE factors might prove different across their study lifecycle. Thirdly, although the studied sample has an adequate number of international companies, the study focused on Oman; by increasing the population to include more countries, new results might emerge and be more generalisable. Fourthly, this research focused on developing countries. Exploring factors affecting SMEs’ sustainability factors in Western countries can help create an understanding of the differences and similarities in various locations. Fifthly, our study used purposive sampling due to the inability to construct a comprehensive sampling frame, thus future research might adopt random sampling for better generalisability. Finally, although 68.9% of our respondents are women, this study did not investigate the gender, size and operated industry as control variables, thus future research might study the role of these variables on sustainable performance.

## Conclusion

This paper contributes to the increasing research in the sustainable performance field for SMEs and adopts the TOE framework factors and resource-based theory view (i.e., green practices) to examine the role of internal green resources on SMEs’ sustainable performance. The study has extended the original TOE framework by adding green practices to measure the SMEs’ sustainable performance. TOE factors underwent conceptualisation as the input, sustainable green practices, such as green HRM, green supply chain, green innovation and green marketing as the process and sustainability as the output. The motivation to integrate this model was to test how sustainable green practices adoption in SMEs can process the TOE factors when affecting sustainable performance. The existing studies on SMEs, however, cover the determinants of the implementation of environmental and social practices, including environment productivity and performance (Rahman & Post, 2012; Revell et al., 2009), environmental and social practices (Chang et al., 2018), social performance (Sutantoputra, 2009) and green innovation (Arnold, 2017). However, no previous study has combined the four mentioned green practices based on the RBV perspective to offer an inclusive theoretical framework for determining how the TOE framework can lead SMEs’ sustainable performance through green practices.

The results of this study provide clear evidence that technological and organisational factors represent crucial inputs compared to environmental factors for green innovation, green HRM and green marketing when SMEs seek sustainable performance. Specifically, a positive strong relationship is shown between technological factors and green innovation, organisational factors and green HRM and environmental factors and green marketing. Accordingly, adopting recruitment policies based on environmental criteria, namely, being environmentally responsible and committed, require SMEs during the pandemic to invest more time and effort in sustainable development, in addition to paying more courtesy to the environmental and societal values connected with sustainable development. Moreover, redesigned production and operation processes to improve environmental efficiency and improved products or services to meet new environmental criteria require higher investment in R & D and the use of updated technology in the production process, service delivery and marketing. During the pandemic, if SMEs try to endorse the internal culture of minimising their emissions, consumption of resources and the environmental impact of their products, they are more likely to use more eco-friendly products and utilise digital communications methods for promoting products and services. Consequently, SMEs can sustain more during times of crisis to reduce hazardous waste, use more environmentally friendly materials, improve environmental compliance and focus on green HRM and green innovation.

## Appendix 1

### Measurement Items

**Table 5:** Measurement items (including those deleted due to low loading value)

Envi1	Our company minimizes its consumption of resources (raw materials, water, and energy)	0.795
Envi2	Our company minimizes its emissions into the air (greenhouse gases and other substances)	0.787
Envi3	Our company minimizes its releases into the water	0.842
Envi4	Our company minimizes residual materials	0.827
Envi5	Our company minimizes the environmental impact of its products	0.782
GHRM1	Our company adhering to select employees based on environmental criteria	0.769
GHRM2	Our company adhering to attract employees through environmental commitment	0.772
GHRM3	Our company adhering to provide environmental training to the employees	0.821
GHRM4	Our company adhering to provide environmental training to the managers	0.816
GHRM5	Job description in our company includes environmental responsibilities	0.798
GHRM6	Our company adhering to involve its employees in environmental issues	0.744
GI1	Our company lower consumption of e.g. water, electricity, gas and petrol during production/use/disposal	0.691
GI2	Our company recycle, reuse, and remanufacture materials or parts	0.779
GI3	Our company use cleaner or renewable technology to make savings (such as energy, water, waste)	0.692
GI4	Our company redesign of production and operation processes to improve environmental efficiency	0.769
GI5	Our company redesign and improve products or services to meet new environmental criteria or directives	0.724
GI6	Our company uses less or non-polluting/toxic materials that are environmentally friendly	0.799
GI7	Our company uses materials that are easy to recycle, reuses, and decompose	0.734
GI8	Our company recovers company's end-of-life products and recycling	0.749
GM1	We encourage the use of e-commerce, because it is more eco-friendly	0.792
GM2	We prefer digital communication methods for promoting our products/services, because it is more eco-friendly	0.768
GM3	We apply a paperless policy in our procurement where possible	0.809
GM4	We use recycled or reusable materials in our products/services	0.771
GM5	We absorb the extra cost of an environmental product/service	0.707
	**Has your company ever taken the following action with your main suppliers or subcontractors?**	
GSC1	Supplier selection on environmental criteria	0.781
GSC2	Advising suppliers on environmental technical issues	0.817
GSC3	Engaging suppliers in product eco-design & development	0.809
GSC4	Appraising environmental performance of the suppliers	0.809
GSC5	Requiring suppliers or subcontractors to obtain a third-party certification of environmental management system (EMS) such as ISO14000	0.789
Orga1	The adoption of sustainable development practices by your company gives you great pride	0.741
Orga2	You care about the environmental and societal values associated with sustainable development	0.804
Orga3	The benefits associated with adopting sustainable development practices are generally greater than the investments they require	0.795
Orga4	The time and effort invested in sustainable development has been beneficial to your business	0.804
Orga5	You believe that it is your duty to favor the adoption of sustainable development practices by your company	0.792
Orga6	Your business environment requires you to adopt sustainable development practices	0.752
Orga7	It is important for you to see your company as having adopted sustainable development practices	0.635
SP1	Our company adhering to reduce paper use	0.627
SP2	Our company adhering to reduce hazardous waste/scrap	0.776
SP3	Our company adhering to reduce in consumption of gasoline/fuel	0.762
SP4	Our company adhering to build partnership with green organizations and suppliers	0.789
SP5	Our company adhering to improve of environmental compliance	0.77
SP6	Our company adhering to use environmental friendly material	0.771
Tech1	Our company invested in R & D to produce quality products	0.729
Tech2	Our company used new technology in the production process	0.754
Tech3	Our company used new methods/procedures in production and service delivery	0.777
Tech4	Our company used new technology in marketing new products	0.778
Tech5	Our company market share has increased due to the use of the new technology in marketing	0.76
Tech6	Using technology, we pay only for what we use	0.721
Tech7	Customization using technology is easy	0.707
Tech8	When we use technology, we find it difficult to integrate the existing work with the web based services	0.639
Tech 9	When we perform many tasks together, using technology, it takes up too much of my time	0.612

## Appendix 2

**Table 6:** Fornell-Larcker

	Envi	GHRM	GI	GM	GSC	Orga	Tech	SP
Envi	**0.807**							
GHRM	0.553	**0.787**						
GI	0.609	0.720	**0.776**					
GM	0.644	0.556	0.579	**0.770**				
GSC	0.609	0.622	0.584	0.592	**0.801**			
Orga	0.630	0.655	0.581	0.605	0.654	**0.790**		
Tech	0.638	0.641	0.713	0.574	0.596	0.652	**0.762**	
SP	0.545	0.680	0.637	0.547	0.491	0.572	0.601	**0.784**

**Table 7:** Cross-loadings matrix

	Envi	GHRM	GI	GM	GSC	Orga	SP	Tech
Envi1	**0.796**	0.457	0.497	0.526	0.439	0.524	0.427	0.488
Envi2	**0.787**	0.418	0.485	0.531	0.452	0.491	0.427	0.463
Envi3	**0.842**	0.418	0.482	0.510	0.476	0.508	0.431	0.566
Envi4	**0.827**	0.456	0.469	0.499	0.514	0.489	0.435	0.504
Envi5	**0.782**	0.479	0.520	0.530	0.565	0.526	0.475	0.548
GHRM1	0.447	**0.769**	0.532	0.440	0.419	0.498	0.556	0.501
GHRM2	0.386	**0.773**	0.467	0.426	0.541	0.537	0.540	0.431
GHRM3	0.410	**0.821**	0.562	0.405	0.538	0.524	0.557	0.480
GHRM4	0.432	**0.816**	0.611	0.462	0.497	0.515	0.572	0.542
GHRM5	0.449	**0.797**	0.593	0.438	0.498	0.527	0.468	0.511
GHRM6	0.485	**0.744**	0.630	0.449	0.448	0.493	0.515	0.555
GI2	0.496	0.556	**0.762**	0.483	0.439	0.508	0.535	0.536
GI4	0.433	0.585	**0.750**	0.435	0.525	0.505	0.515	0.521
GI5	0.438	0.569	**0.752**	0.426	0.379	0.428	0.450	0.546
GI6	0.489	0.565	**0.825**	0.428	0.467	0.453	0.507	0.594
GI7	0.507	0.500	**0.778**	0.470	0.450	0.408	0.433	0.544
GI8	0.473	0.580	**0.790**	0.455	0.456	0.396	0.520	0.580
GM1	0.561	0.473	0.451	**0.792**	0.523	0.533	0.451	0.483
GM2	0.489	0.415	0.406	**0.769**	0.398	0.500	0.421	0.444
GM3	0.476	0.418	0.443	**0.809**	0.415	0.433	0.397	0.394
GM4	0.493	0.429	0.499	**0.771**	0.485	0.439	0.420	0.439
GM5	0.450	0.397	0.432	**0.707**	0.450	0.411	0.413	0.443
GSC1	0.513	0.454	0.408	0.419	**0.780**	0.496	0.377	0.428
GSC2	0.513	0.452	0.444	0.460	**0.818**	0.476	0.377	0.516
GSC3	0.478	0.535	0.493	0.487	**0.810**	0.541	0.421	0.498
GSC4	0.526	0.542	0.530	0.514	**0.811**	0.600	0.416	0.501
GSC5	0.399	0.505	0.454	0.489	**0.787**	0.497	0.371	0.437
Orga1	0.461	0.506	0.464	0.458	0.516	**0.749**	0.437	0.557
Orga2	0.502	0.546	0.463	0.474	0.534	**0.822**	0.438	0.524
Orga3	0.524	0.537	0.455	0.449	0.531	**0.799**	0.473	0.540
Orga4	0.516	0.514	0.471	0.472	0.557	**0.818**	0.467	0.513
Orga5	0.452	0.525	0.447	0.486	0.523	**0.806**	0.455	0.487
Orga6	0.531	0.473	0.452	0.530	0.431	**0.740**	0.440	0.465
SP2	0.430	0.518	0.499	0.409	0.375	0.396	**0.778**	0.458
SP3	0.398	0.499	0.466	0.418	0.332	0.415	**0.755**	0.430
SP4	0.432	0.561	0.525	0.400	0.371	0.442	**0.812**	0.481
SP5	0.472	0.515	0.496	0.478	0.400	0.492	**0.787**	0.494
SP6	0.405	0.570	0.509	0.438	0.439	0.491	**0.787**	0.491
Tech1	0.463	0.510	0.545	0.421	0.431	0.496	0.466	**0.747**
Tech2	0.464	0.505	0.549	0.438	0.524	0.462	0.405	**0.757**
Tech3	0.529	0.481	0.565	0.448	0.464	0.488	0.504	**0.804**
Tech4	0.544	0.520	0.594	0.472	0.436	0.552	0.526	**0.796**
Tech5	0.490	0.459	0.519	0.453	0.445	0.536	0.412	**0.785**
Tech6	0.421	0.457	0.528	0.422	0.460	0.450	0.410	**0.725**
Tech7	0.488	0.486	0.499	0.406	0.415	0.493	0.483	**0.717**

**Table 8:** HTMT

	Envi	GHRM	GI	GM	GSC	Orga	Tech	SP
Envi								
GHRM	**0.633**							
GI	0.701	**0.824**						
GM	0.757	0.650	**0.684**					
GSC	0.699	0.716	0.672	**0.698**				
Orga	0.722	0.747	0.664	0.706	**0.748**			
Tech	0.729	0.729	0.816	0.670	0.682	**0.742**		
SP	0.636	0.789	0.743	0.653	0.573	0.663	**0.697**

**Table 9:** VIFs (inner), and R^2^

			Multicollinearity test (VIF inner)				Model’s predictive accuracy	
Constructs	GHRM	GI		GM	GSC	SP	R^2^	Adj R^2^
Envi	1.948	1.948		1.948	1.948			
GHRM						2.422	0.515	0.513
GI						2.354	0.556	0.554
GM						1.793	0.494	0.492
GSC						1.948	0.51	0.508
Orga	2.008	2.008		2.008	2.008			
Tech	2.043	2.043		2.043	2.043			
SP							0.529	0.526
